# Deciphering the Neural Control of Sympathetic Nerve Activity: Status Report and Directions for Future Research

**DOI:** 10.3389/fnins.2017.00730

**Published:** 2017-12-22

**Authors:** Susan M. Barman, Bill J. Yates

**Affiliations:** ^1^Department of Pharmacology and Toxicology, Michigan State University, East Lansing, MI, United States; ^2^Department of Otolaryngology, University of Pittsburgh, Pittsburgh, PA, United States; ^3^Department of Neuroscience, University of Pittsburgh, Pittsburgh, PA, United States

**Keywords:** sympathetic nerve activity, baroreceptor reflex, central command, exercise pressor response, vestibulosympathetic reflex

## Abstract

Sympathetic nerve activity (SNA) contributes appreciably to the control of physiological function, such that pathological alterations in SNA can lead to a variety of diseases. The goal of this review is to discuss the characteristics of SNA, briefly review the methodology that has been used to assess SNA and its control, and to describe the essential role of neurophysiological studies in conscious animals to provide additional insights into the regulation of SNA. Studies in both humans and animals have shown that SNA is rhythmic or organized into bursts whose frequency varies depending on experimental conditions and the species. These rhythms are generated by brainstem neurons, and conveyed to sympathetic preganglionic neurons through several pathways, including those emanating from the rostral ventrolateral medulla. Although rhythmic SNA is present in decerebrate animals (indicating that neurons in the brainstem and spinal cord are adequate to generate this activity), there is considerable evidence that a variety of supratentorial structures including the insular and prefrontal cortices, amygdala, and hypothalamic subnuclei provide inputs to the brainstem regions that regulate SNA. It is also known that the characteristics of SNA are altered during stress and particular behaviors such as the defense response and exercise. While it is a certainty that supratentorial structures contribute to changes in SNA during these behaviors, the neural underpinnings of the responses are yet to be established. Understanding how SNA is modified during affective responses and particular behaviors will require neurophysiological studies in awake, behaving animals, including those that entail recording activity from neurons that generate SNA. Recent studies have shown that responses of neurons in the central nervous system to most sensory inputs are context-specific. Future neurophysiological studies in conscious animals should also ascertain whether this general rule also applies to sensory signals that modify SNA.

## Introduction

The 2009 report (Schlaich et al., 2009) demonstrating that catheter-based radiofrequency renal denervation could reverse the elevated levels of blood pressure and muscle sympathetic nerve activity (MSNA) in a cohort of hypertensive patients with end stage kidney failure drew considerable attention to the role of sympathetic dysfunction in cardiovascular disease. Disturbances in SNA are thought to contribute to the genesis and/or the maintenance of many cardiovascular diseases including essential hypertension, heart failure, orthostatic hypotension, and psychogenic heart disease or heart disease that is a consequence of psychiatric disorders (Mathias, 1996; Benarroch, 1997; Klein et al., 2003; Low and Engstrom, 2012; Martínez-Martínez et al., 2014; Wehrwein and Barman, 2014). As reviewed by Wehrwein et al. (2016), several neurological diseases/disorders have imbalances in SNA as either a direct cause of the disease (e.g., multiple system atrophy, Shy Drager syndrome, pure autonomic failure) or as a consequence of a disease (e.g., Parkinson disease). The therapeutic effects of many commonly used prescription and over-the-counter drugs result from modulation of sympathetic function; examples include β-adrenoceptor antagonists for heart failure, hypertension, and glaucoma; β-adrenoceptor agonists for asthma and emphysema, α-adrenoceptor antagonists for benign prostatic hyperplasia, and α-adrenoceptor agonists to dilate pupils for ophthalmic exams (see review by Esler, 2012). Thus, in order to appreciate fully integrative physiology and pathophysiology, we need to be able to measure SNA and to understand how it can be modulated in different behavioral states and under pathophysiological conditions.

This review summarizes our current state of knowledge of the central nervous system mechanisms that generate and modulate SNA. A major focus is gaps in our knowledge about these neural mechanisms, and the strengths and weaknesses of the experimental paradigms that have been used to decipher the control of autonomic function. In particular, we will discuss the promise of evolving techniques for examining the neural control of SNA and cardiovascular function in conscious animal models.

## Indirect indices of SNA

During the past several decades, investigators have used a variety of approaches to assess “sympathetic tone,” including indirect measures such as pharmacological or surgical blockade of autonomic ganglia (King et al., 2007; Yoshimoto et al., 2010a), evaluation of the range of fluctuations of blood pressure (blood pressure variability) over time (Parati et al., 2013), changes in the frequency components of heart rate or systolic blood pressure variability (see reviews by Acharya et al., 2006; Reyes del Paso et al., 2013), and a measure of total or regional norepinephrine spillover via the use of radiotracer dilution technology (Esler et al., 1984). Several reviews (Guild et al., 2010; Malpas, 2010; Charkoudian and Wallin, 2014) provide an excellent critique of the pros and cons of each of these indirect methods in establishing information about sympathetic control of the cardiovascular system. Whereas each of these methods gives us some important clues about sympathetic function, none can actually substitute for a direct recording of the activity within the sympathetic nerves that control various autonomic effector organs. In 1932 Adrian and colleagues were the first to publish a recording of the naturally occurring activity in sympathetic nerve fibers (cervical and abdominal) in anesthetized cats and rabbits (Adrian et al., 1932). About 36 years later, Karl-Erik Hagbarth pioneered the use of microneurography to record MSNA in humans by inserting a needle into his own ulnar nerve (see Vallbo et al., 2004).

## Rhythms: the hallmark of SNA

**Figure 1:**
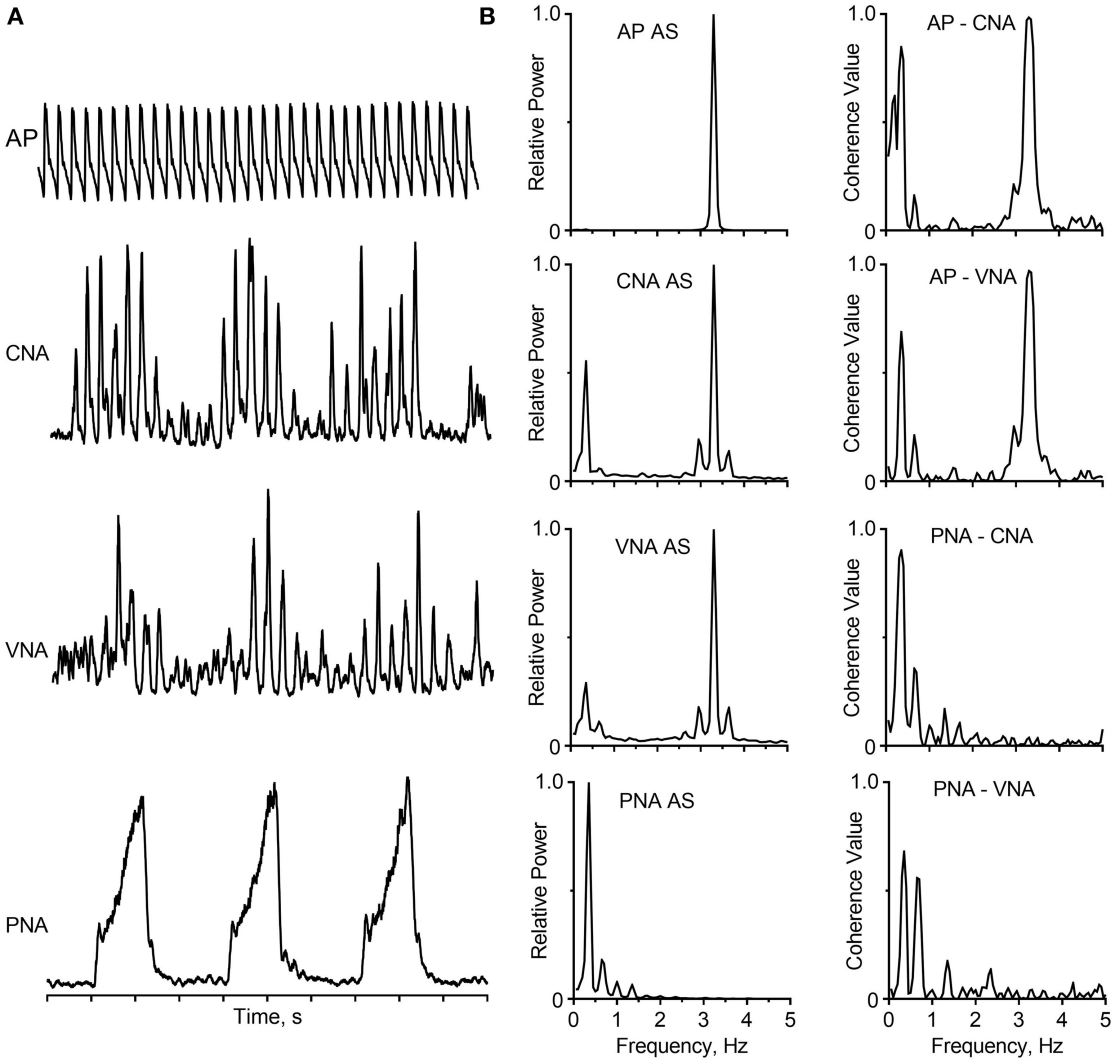
Cardiac-related and respiratory activity recorded from two branches of the left stellate ganglion in a barbiturate-anesthetized, paralyzed, and artificially-ventilated cat. **(A)** Traces (*top* to *bottom*) show the arterial pressure (AP), cardiac nerve activity (CNA), vertebral nerve activity (VNA), integrated phrenic nerve activity (PNA), and time base (1 s/division). The capacity-coupled preamplifier bandpass setting was 30–3,000 Hz (CNA, VNA) or 10–1,000 Hz (PNA). Signals were passed through a 50/60 Hz noise eliminator (Hum Bug; Quest Scientific) and a moving averager (CWE, Model MA-821RSP) with a 50-ms (CNA, VNA) or 100-ms (PNA) time constant. **(B)** Autospectra (left) and coherence functions (right) for these signals. Spectra are based on 35 20-s windows with 50% overlap, and they have a frequency resolution of 0.05 Hz per bin. Data showing cardiac- and respiratory-related rhythms appear in CNA and VNA have been published (Barman and Kenney, 2007; Barman, 2016); this figure has been created *de novo* but does not contain any original data.

Following the landmark studies by Adrian et al. (1932) and Vallbo et al. (2004), many investigators have placed recording electrodes in or on sympathetic nerves supplying a variety of target organs, including the heart, kidney, splanchnic circulation, skeletal muscle vasculature, brown adipose tissue, spleen, and skin. Recordings of SNA have been obtained using multiple experimental models including barbiturate-, chloralose-, or urethane-anesthetized cats, rabbits, and rodents, decerebrate-unanesthetized cats and rodents, isolated rodent brainstem-spinal preparations, and conscious cats, rabbits, rodents, sheep, and human subjects (see reviews by Barman and Gebber, 2000; Vallbo et al., 2004; Wallin and Charkoudian, 2007; Guild et al., 2010; Malpas, 2010; Kenney and Mosher, 2013; Charkoudian and Wallin, 2014; White et al., 2015; Hart et al., 2017). A common feature of these diverse studies is that bursts of SNA are synchronized to the phases of the cardiac cycle (cardiac-related activity) as a result of baroreceptor-induced entrainment. In addition, the amplitude of these cardiac-related bursts waxes and wanes on the time scale of the respiratory cycle (respiratory-related activity), reflecting central and reflex-induced cardiorespiratory synchronization. These features of SNA are illustrated by the data in Figure 1 from a cat that was anesthetized with a mixture of diallybarbiturate and urethane, paralyzed with gallamine triethiodide, and artificially ventilated. The traces show arterial pressure (AP), inferior cardiac (to the heart) and vertebral (to the vasculature of the skeletal muscle of the forelimb) nerve activity (CNA, VNA), and phrenic nerve activity (PNA). The cardiac-related and respiratory-related bursts of SNA are evident in the raw recordings from two functionally distinct sympathetic nerves emanating from the left stellate ganglion (Figure 1A); also these hallmark characteristics of SNA can be quantified by spectral analysis using fast Fourier transform (Figure 1B). The autospectra of CNA and VNA show peaks at both the frequency of the central respiratory cycle and at the frequency of the heartbeat. Coherence analysis showed that these components of SNA were strongly correlated to PNA and the AP, respectively.

Since the cardiac- and respiratory-related bursts of SNA are prominent in recordings from most sympathetic nerves in mammalian species, they (especially the cardiac-related rhythm) are regarded as the hallmark of SNA. In fact, when using microneurography to record MSNA in human subjects, the appearance of the cardiac-related activity signals to the investigator that the recording electrode has reached the appropriate target. Nonetheless, central sympathetic circuits are dynamic and can generate different burst patterns depending on the physiological state, the type of nerve being studied, as well as the species (Malpas, 1998, 2010; Chang et al., 1999; Hashimoto et al., 1999; Barman and Gebber, 2000; Barman and Kenney, 2007; Charkoudian and Wallin, 2014). For example, cardiac- and respiratory-related rhythms are typically absent in the activity recorded from cutaneous vasoconstrictor fibers, sudomotor fibers, epinephrine-regulating adrenal preganglionic neurons, and nerves supplying the brown adipose tissue (Jänig et al., 1983; Johnson and Gilbey, 1994; Macefield and Wallin, 1996; Morrison, 1999; Morrison and Cao, 2000). Differences in neuronal activity patterns among a wide population of sympathetic nerves may reflect the non-uniform influences of central and peripheral inputs to sympathetic outflow (Morrison, 2001). In addition to the cardiac- and respiratory-related periodicities, oscillations ranging from ~0.04 Hz to at least 10-Hz have been recorded from sympathetic nerves in a variety of species (see reviews by Malpas, 1998, 2010; Barman and Gebber, 2000; Barman, 2016).

## Comparing various experimental models for recording SNA

While the number of laboratories with expertise in recordings of SNA from human subjects has increased in the twenty-first century, animal models remain the mainstay in central autonomic research. Table 1 summarizes some of the benefits and limitations of using human subjects and anesthetized or conscious animal models to study sympathetic neural control of autonomic function. In addition to these models which are the focus of this review article, the authors acknowledge that work on reduced preparations such as the isolated brainstem-spinal cord preparation, working heart-brain preparation, and the decerebrate, artificially-perfused rat preparation have contributed to our base of knowledge regarding the peripheral and central control of the autonomic nervous system (Paton, 1996; Pickering and Paton, 2006; Chen et al., 2011).

**Table 1:** Benefits and limitations of recording SNA in different models.

**Benefit/Limitation**	**Human**	**Conscious animal**	**Anesthetized animal**
No question regarding relevance of research	^*^		
Reproducible recordings over extended periods of time	^*^	^*^	
Study SNA in different disease models, with and without treatment	^*^	^*^	^*^
Record SNA long term in same subject to track disease development and progression		^*^	
Study state-dependent and behavior-induced changes in SNA	^*^	^*^	
Record from visceral sympathetic nerves		^*^	^*^
Record from many nerves simultaneously to study differential control of SNA			^*^
Only MSNA and SSNA can be recorded	^*^		
Integrity of baroreceptor reflex is needed to verify recording is from a sympathetic nerve	^*^		
Manipulate central sympathetic circuits pharmacologically		^*^	^*^
Record simultaneously from central neurons and sympathetic nerves		^*^	^*^
Neuromuscular blockade can be used to avoid movement artifacts			^*^
Anesthesia can alter cardiovascular stability and makes the preparation less physiological			^*^
Telemetry is in its infancy; very few labs capable of doing this		^*^	
Less than 50% success rate in valid nerve recordings		^*^	

* *Denotes that the listed characteristic relates to this model*.

No doubt the ideal experimental model to study changes in SNA in health and disease in the human population is to record SNA in human subjects. As articulated in several recent reviews (Wallin and Charkoudian, 2007; Charkoudian and Wallin, 2014; White et al., 2015; Hart et al., 2017), MSNA burst frequency or burst incidence in a supine individual is reproducible in recordings made many months apart if his/her physiological status has not changed drastically. As cautioned by Hart et al. (2017) this is the case as long as recording conditions are standardized (e.g., room temperature between 21 and 24°C, subject at rest but not sleeping, and room noise at a minimum). However, MSNA burst frequency increases with age and during exposure to high altitudes; and burst incidence is higher in individuals with various cardiovascular pathologies such as chronic renal failure, congestive heart failure, diabetes, hypertension, metabolic syndrome, obesity, and obstructive sleep apnea (Wallin and Charkoudian, 2007; Charkoudian and Wallin, 2014; White et al., 2015; Hart et al., 2017).

Despite the recognized scientific and practical value to recording SNA in human subjects, one cannot design experiments using human subjects to study changes in MSNA before, during, and after the development of certain pathologies as one cannot readily predict which subjects would qualify for entry into the study. Also, the time (years) needed to complete such a study induces other “expected” age-related changes in MSNA (Wallin and Charkoudian, 2007; Charkoudian and Wallin, 2014). Another limitation of studies in human subjects is that one cannot intentionally manipulate regions of the central nervous system to assess the impact of such on SNA. Also, one cannot simultaneously record from individual brainstem neurons and sympathetic nerves in an effort to understand the neural pathways involved in regulating SNA. Perhaps even more problematic, one cannot record from a visceral (e.g., cardiac, splanchnic, or renal) nerve in a human subject and yet these may be the most important nerves to study in terms of the basis for cardiovascular disease or dysfunction (Osborn and Fink, 2010). For example, Osborn and Fink (2010) have data supporting the view that splanchnic SNA is increased, renal SNA is decreased, and muscle SNA is unchanged during angiotensin II-induced hypertension in rats.

**Figure 2:**
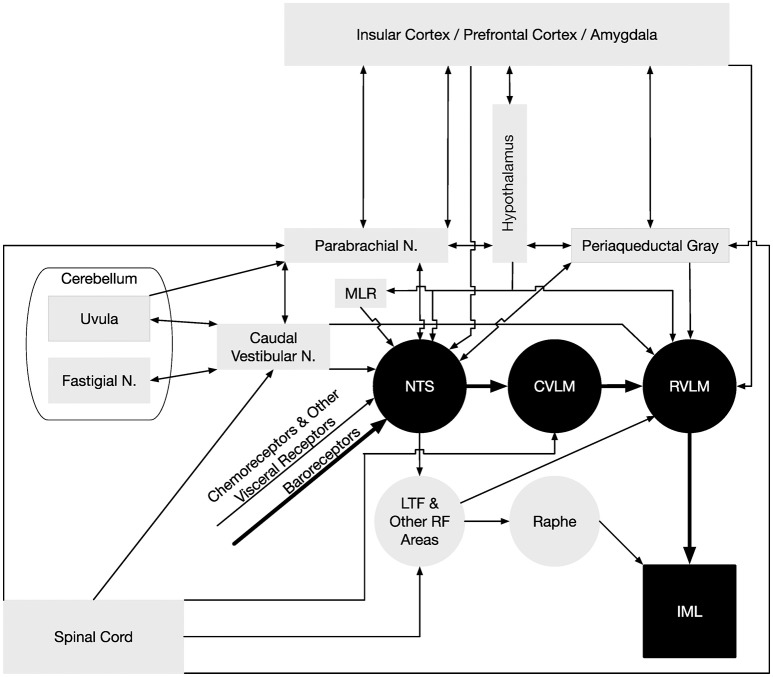
Neural pathways that regulate blood pressure. The minimal “textbook” pathway that produces baroreceptor reflexes is denoted using black-filled symbols and thick arrows, and consists of neurons in the nucleus tractus solitarius (NTS) that receive baroreceptor inputs, interneurons in the reticular formation of the caudal ventrolateral medulla (CVLM), bulbospinal neurons in the rostral ventrolateral medulla (RVLM), and sympathetic preganglionic neurons located in the intermediolateral cell column (IML) of the thoracic and upper lumbar spinal cord. However, many other neural structures and pathways participate in regulating sympathetic nervous system effects on the control of blood pressure, which are indicated using gray-filled symbols and thin arrows. The medullary raphe nuclei act in concert with the RVLM in adjusting sympathetic nervous system outflow to the cardiovascular system (Barman and Gebber, 2000). Both the RVLM and raphe nuclei receive substantial inputs through particular regions of the reticular formation (RF), including the lateral tegmental field (LTF) (Barman and Gebber, 1987, 1989). In addition to baroreceptor inputs, a variety of other visceral inputs including those from chemoreceptors contribute to regulating sympathetic nervous system activity (Thorén et al., 1976; Guyenet, 2000), as do somatic signals relayed from the spinal cord (Wilson and Hand, 1997; Boscan et al., 2002) and vestibular system (Yates et al., 2014). Somatic signals are conveyed to the RVLM through the CVLM and other regions of the reticular formation (Masuda et al., 1992; Steinbacher and Yates, 1996a,b), the parabrachial nucleus and periaqueductal gray (Balaban, 1996; Andrew, 2010), the caudal portions of the vestibular nuclei (Holstein et al., 2011a), and regions of the cerebellum (uvula, fastigial nucleus) (Nisimaru, 2004; Yates et al., 2014). Cerebellar influences on the control of blood pressure are mediated in part through connections with parabrachial neurons that project to NTS (Bradley et al., 1991). Several midbrain regions participate in regulating blood pressure by providing inputs to NTS and RVLM, including the periaqueductal gray (Lovick, 1993), parabrachial nucleus (Saper and Loewy, 1980; Hamilton et al., 1981; Mraovitch et al., 1982; Felder and Mifflin, 1988; Herbert et al., 1990; Mifflin and Felder, 1990; Paton et al., 1990; Krukoff et al., 1993), and mesencephalic locomotor region (MLR) (Degtyarenko and Kaufman, 2005). The MLR regulates locomotion in some species, and projections from the MLR to NTS likely change the set point of the baroreceptor reflex during locomotion (Degtyarenko and Kaufman, 2005). Hypothalamic nuclei (Ross et al., 1981; Berk and Finkelstein, 1982; Kannan and Yamashita, 1983; van der Kooy et al., 1984; Jordan et al., 1988; Mifflin et al., 1988; Wible et al., 1988; Mifflin and Felder, 1990; Markgraf et al., 1991; Allen and Cechetto, 1992; Cechetto and Chen, 1992; Martin and Haywood, 1992; Ebihara et al., 1993; Martin Haywood and Haywood, 1993; Kawano and Masuko, 1995; Badoer, 1998; Coote et al., 1998; Fontes et al., 2001; Cravo et al., 2003; Horiuchi et al., 2006; Kawabe et al., 2008; Bowman et al., 2013; Sapru, 2013) provide inputs to NTS and/or the RVLM, as do the amygdala (Kapp et al., 1982; Schwaber et al., 1982; van der Kooy et al., 1984; Saha, 2005; Saha et al., 2005; Bowman et al., 2013), and prefrontal and insular cortices (Shipley, 1982; van der Kooy et al., 1984; Cechetto and Chen, 1990, 1992; Verberne and Owens, 1998; Owens and Verberne, 2000; Gabbott et al., 2005; Sévoz-Couche et al., 2006). Inputs from the telencephalon to the RVLM and NTS are both direct and indirect through relays in the hypothalamus, periaqueductal gray, and parabrachial nucleus (Saper and Loewy, 1980; Cechetto and Chen, 1990, 1992; Krukoff et al., 1993; Hardy, 1994).

It is not surprising that many autonomic neuroscientists have relied on animal models to study SNA and the central neural control of cardiovascular function. Studies using anesthetized or decerebrate animals are amenable to recording simultaneously sympathetic outflow to multiple effector organs and the activity of central neurons. Also this preparation is well-suited for recording changes in SNA and blood pressure produced by elicitation of reflexes and by chemical activation or inactivation of various brain regions (see reviews by Dampney, 1994; Malpas, 1998, 2010; Barman and Gebber, 2000; Guyenet, 2000; Guild et al., 2010; Kenney and Mosher, 2013). Such studies have provided us with a wealth of information on the roles of various peripheral and central regions involved in the control of SNA and cardiovascular function. They also have allowed us to gain an appreciation for the complexity of autonomic regulation including differential control of regional sympathetic outflow. For example, Barman and Gebber and their colleagues have used several approaches in anesthetized cats to identify central neurons that generate and/or transmit rhythmic activity to the spinal intermediolateral cell column (IML) that contains the cell bodies of preganglionic neurons (Barman and Gebber, 1992, 1993, 1997, 1998, 2007; Barman et al., 1994, 1995, 1997, 1999, 2002, 2005; Orer et al., 1999, 2008). These experimental approaches include (1) applying correlation analyses (spike-triggered averaging and coherence analysis) to the simultaneously recorded activity of individual brainstem neurons and sympathetic nerves, (2) microinjecting agonists or antagonists of putative central neurotransmitters, including glutamate, GABA, serotonin, and catecholamines, into different medullary and pontine regions to characterize changes in SNA rhythmicity, and (3) using the technique of antidromic activation to determine interconnections of medullary neurons and projections of brainstem neurons to the IML. Studies using anesthetized animals have also provided us with a wealth of information on the complexity of the neurochemistry of central autonomic pathways (Benarroch, 1997; Stornetta, 2009). Knowing the specific neuronal phenotypes of central neurons has allowed for the application of appropriate optogenetic and pharmacogenetic actuators to identify the roles of specific groups of neurons in the control of SNA and blood pressure (Guyenet, 2006; Wenker et al., 2017). These studies along with others (see reviews by Dampney, 1994; Barman and Gebber, 2000; Guyenet, 2000; Barman, 2016) have been a part of the framework for constructing the wiring diagram shown in Figure 2 that depicts the central pathways that regulate the cardiovascular system; this figure is discussed in more detail below.

Amongst the major limitations of an anesthetized animal preparation is the fact that anesthesia alters blood pressure and respiration, two major factors that modulate SNA, as well as having direct effects on SNA (Shimokawa et al., 1998; Neukirchen and Kienbaum, 2008). The effect of anesthetics on the activity of neurons that control SNA is additionally problematic, and one cannot study the impact of behavioral or state-dependent changes in SNA. Of course, the inability to do long-term recordings of SNA in anesthetized animals limits what information we can gain about the role of changes in SNA in disease development or progression. Instead, when recording SNA of anesthetized animals, one needs to compare levels of SNA from different groups of animals (e.g., a normotensive group and a hypertensive group).

Some of the limitations associated with the use of anesthetized animal models can be overcome by performing long-term recordings of SNA in conscious, freely behaving animals. There are two major approaches to these chronic nerve recordings. One can use a tethered system in which wires attached to a recording electrode on a nerve are exteriorized and connected to a recording device (e.g., Yoshimoto et al., 2010b; Hamza and Hall, 2012) or one can use a telemetry-based implantable nerve amplifier (e.g., Barrett et al., 2003; Guild et al., 2012; Muntzel et al., 2012; Stocker and Muntzel, 2013). Twenty-first century advancements in continuous nerve recording techniques have not only eliminated the influence of anesthesia on the measured variables, but they have allowed researchers to study SNA in the same animal before, during, and after development of a pathology or before and during a change in behavior (e.g., sleep, exercise, stress) or change in diet (e.g., high salt or high fat diet). The field of central autonomics has benefited by gaining new information regarding the contribution of changes in SNA in health and disease (see reviews by Guild et al., 2010; Wehrwein and Barman, 2014; Hart et al., 2017). Wehrwein and Barman (2014) recently highlighted several studies that have used continuous (up to 21 days) nerve recordings to determine the time course of changes in SNA as hypertension develops. It seems that very few studies have been able to show unequivocally that an increase in SNA underlies the development or maintenance of hypertension.

Few laboratories have mastered the ability to simultaneously record from two sympathetic nerves in conscious animals, limiting the ability to use these conscious animal models to study the critical issue of differential control of SNA. Notable exceptions include Miki and colleagues who have made notable contributions to this field by the use of recordings of the activity more than one sympathetic nerve often in conjunction with vascular responses in rats under a variety of conditions, including rapid eye movement (REM) and non-REM sleep and exercise (Miki and Yoshimoto, 2005, 2010). For example, they showed that at the transition between non-REM and REM sleep, there is a decrease in renal SNA with an increase in renal blood flow and an increase in lumbar SNA with a decrease in hindlimb blood flow.

Other drawbacks of recording from SNA in conscious animals is the inability to pair these recordings with sophisticated approaches like simultaneous recording of central sympathetic neurons and using antidromic activation to target specific types of neurons (example, neurons projecting to the IML). In fact, it is our understanding that recording from central sympathetic neurons in a conscious animal model has been mastered only by Yates et al. (Barman et al., 2011; DeStefino et al., 2011). These studies are discussed below.

Regardless of the experimental model to be chosen (human subjects or instrumented animals), it is important to be adequately trained in the technologies to be used. Recent reviews such as those by Guild et al. (2010) and Hart et al. (2017) articulate the challenges in gaining this expertise, including care in handling nerves, waiting adequate time between surgery or other manipulations and the beginning of the recording period, and taking care to eliminate movement-induced and electrical artifacts. Since there is no perfect experimental model, one needs to select the model that best addresses the questions at hand. For example, if the question relates to the impact of activation or deactivation of brain regions on SNA, the best model may be anesthetized animals (Masuda et al., 1992; Barman et al., 1994, 2002, 2005; Orer et al., 2008; Barman and Gebber, 2009). When one is interested in identifying central neurons involved in control of SNA, decerebrate, anesthetized, or conscious animal models can be utilized, with the caveat that the firing patterns of the neurons can be altered by anesthesia or decerebration (Barman and Gebber, 1992, 1997; Barman et al., 2011; DeStefino et al., 2011).

## Contributions of the midbrain, diencephalon, and telencephalon in controlling SNA and blood pressure

Most studies deciphering the neural control of SNA and blood pressure have focused on the brainstem. This is largely due to the fact that the essential neurons for controlling cardiac-related fluctuations in SNA and mediating the baroreceptor reflex are located in the brainstem and spinal cord (Dampney, 1994). Across species, the key circuit that mediates the baroreceptor reflex includes neurons in the nucleus of the tractus solitarius (NTS) and caudal and rostral ventrolateral medulla (CVLM, RVLM). As shown in Figure 2, neurons in the RVLM convey the integrated brainstem signal to sympathetic preganglionic neurons located in the IML. This basic circuit for regulating baroreceptor-mediated changes in SNA is widely represented in textbooks, such that the pathway (in addition to connections from NTS to parasympathetic neurons that adjust heart rate) is sometimes represented as the totality of neural control of cardiac function.

The baroreceptor reflex, as assessed by considering a variety of responses (e.g., changes in SNA or heart rate) to stimulation of baroreceptors, is qualitatively similar in conscious, anesthetized, and decerebrate preparations of a variety of species (Seagard et al., 1982, 1983; Abdel-Rahman et al., 1987; Stornetta et al., 1987; Matsukawa and Ninomiya, 1989; Suzuki et al., 1993; Farber et al., 1995; Muzi and Ebert, 1995; Ebert et al., 1998; Katsuda et al., 2000; Lee et al., 2004). Such observations reinforce the notion that the control of SNA is mainly a function of the brainstem.

Although the baroreceptor reflex is qualitatively the same across experimental preparations, it is also recognized that the dynamic properties of the response are altered by anesthetics and decerebration (Seagard et al., 1982, 1983; Abdel-Rahman et al., 1987; Stornetta et al., 1987; Matsukawa and Ninomiya, 1989; Suzuki et al., 1993; Farber et al., 1995; Muzi and Ebert, 1995; Ebert et al., 1998; Katsuda et al., 2000; Lee et al., 2004). The baroreceptor reflex is also affected by transitions in sleep/wake and behavioral states and following stress (Stephenson et al., 1981; Coote, 1982; Conway et al., 1985; Del Bo et al., 1985; Knuepfer et al., 1986; Kasting et al., 1987; Mion and Krieger, 1988; Sei et al., 1994; Vaile et al., 1996; Sei and Morita, 1999; Zoccoli et al., 2001; Kanbar et al., 2007; Grippo et al., 2008; Julien, 2008; Cortelli et al., 2012; Almeida et al., 2014; Kuo et al., 2014). The latter findings highlight an influence of supratentorial brain regions on the brainstem circuitry that regulates SNA and blood pressure.

A variety of approaches, including neuroanatomical studies and neurophysiological experiments using microstimulation of brain regions and/or antidromic stimulation, have shown that a number of structures in the midbrain, diencephalon, and telencephalon affect the activity of neurons in NTS and the RVLM (Verberne et al., 1997). These structures are indicated in Figure 2, and include the parabrachial nucleus, periaqueductal gray, several hypothalamic nuclei, amygdala, insula, and prefrontal cortex. One study in anesthetized animals showed that elimination of forebrain inputs caused a precipitous change in SNA (Huang et al., 1987), highlighting the potential significance of supratentorial regions in the control of blood pressure. In addition, microneurography studies in humans established that mental stress results in increases in MSNA (Anderson et al., 1991; Callister et al., 1992; Carter et al., 2005; Carter and Lawrence, 2007; Carter and Ray, 2009). Considering the connections and functions of structures such as the insula, prefrontal cortex, and amygdala that provide inputs to the RVLM and NTS, a reasonable hypothesis is that they contribute to adjusting SNA during stress and affective responses (Verberne et al., 1997). However, there is no direct evidence to support this hypothesis, and the required experiments would require the use of a conscious animal preparation, since the complex signal integration that occurs in the telencephalon is profoundly altered by anesthesia and eliminated by decerebration.

## Movement-related changes in SNA

Movement requires changes in SNA in order to meet the metabolic needs of an individual. Two examples of movement-related increases in SNA are well-documented: (1) those that occur during exercise, which are accompanied by resetting of the baroreceptor reflex (Waldrop et al., 1996; Williamson, 2010; Fadel and Raven, 2012; Matsukawa, 2012; Mitchell, 2012) and (2) those that occur during movements that lead to peripheral blood pooling, such as standing from a supine position in humans (Yates et al., 2014). These two responses are distinct and will be discussed separately below.

### Exercise-related changes in SNA

In both animals and humans, adjustments in SNA and alterations in the set-point of the baroreceptor reflex are initiated when exercise begins (Waldrop et al., 1996; Fadel and Raven, 2012). The changes in the baroreceptor set-point are needed to allow blood pressure to increase during exercise. The term “central command” refers to feedforward changes in autonomic nervous system activity that accompany muscle contraction. In decerebrate or anesthetized cats, stimulation of regions of the lateral and caudal hypothalamus, fields of Forel, mesencephalic locomotor region, and midbrain ventral tegmental area elicit parallel changes in motor activity and cardiovascular responses (Waldrop et al., 1996; Nakamoto et al., 2011; Matsukawa, 2012). However, little is known about signal processing in these regions that leads to changes in SNA and the baroreceptor reflex set point, as the required experiments would require the use of an awake, behaving animal preparation. The changes in the baroreceptor reflex during exercise are due at least in part to inhibitory neurotransmission in NTS (Degtyarenko and Kaufman, 2005; Potts, 2006).

In addition to central command, inputs from group III and group IV muscle afferents that respond to mechanical and chemical stimuli trigger changes in SNA and blood pressure (Kaufman, 2012). This response is often referred to as the “exercise pressor reflex,” and indicates when blood perfusion is not adequate to meet metabolic needs. The exercise pressor reflex is mediated at least partly through brainstem circuitry, and the neural mechanisms of the response have been investigated extensively in anesthetized and decerebrate animals. Spinoreticular pathways convey muscle afferent signals to NTS and the ventrolateral medulla, and these relatively direct connections with brainstem areas that control SNA are believed to trigger the exercise pressor response (Stornetta et al., 1989; Masuda et al., 1992; Potts, 2001, 2006; Degtyarenko and Kaufman, 2002; Wilson et al., 2002). There is also some evidence that central command and the exercise pressor reflex are at least partially synergistic (Gallagher et al., 2006; Michelini et al., 2015), although the combined influences of the two on activity of neurons in NTS and the RVLM are yet to be determined.

### Posture-related changes in SNA

Head up movements, such as standing in humans, can result in decreased return of blood to the heart and orthostatic hypotension (Wieling and von Lieshout, 1993; Mano, 2001). During such movements, SNA must increase to augment vascular resistance and decrease lower body blood flow to maintain stable blood pressure (Rushmer, 1976; Wieling and von Lieshout, 1993; Mano, 2001). Unloading of baroreceptors during head-up movements undoubtedly plays a role in adjusting SNA should blood pressure decrease. However, many lines of evidence (reviewed in Yates et al., 2014) show that sensory inputs from the vestibular system also play an important role in adjusting SNA during postural adjustments. The caudal portion of the vestibular nucleus complex provides direct inputs to RVLM, as well as indirect inputs that are conveyed through the reticular formation (Steinbacher and Yates, 1996a,b; Holstein et al., 2011a,b, 2014). In human subjects, movements of the head that activate vestibular receptors produce a large increase in MSNA (Hume and Ray, 1999). In conscious animals, bilateral labyrinthectomies attenuate the increase in vascular resistance that ordinarily occurs in the hindlimbs during head-up tilts (Wilson et al., 2006; Yavorcik et al., 2009).

Although vestibular-elicited changes in SNA can be demonstrated in decerebrate, anesthetized, and conscious animals, the properties of the responses differ considerably between experimental preparations (Yates et al., 2014). Direct comparisons of response characteristics have been made in decerebrate and conscious animals. In decerebrate cats, the activity of ~50% of RVLM neurons, including those with baroreceptor inputs, was modulated by 10° tilts (DeStefino et al., 2011). Activation of vestibular receptors by 10–15° head-up tilts also produced appreciable increases in SNA (Yates and Miller, 1994). However, in conscious cats, only 1% of RVLM neurons responded to 10–15° rotations (DeStefino et al., 2011), which elicited no appreciable vasoconstriction (Wilson et al., 2006; Yavorcik et al., 2009). These data show that in decerebrate animals, non-physiologic (exaggerated) increases in sympathetic nerve activity occur during head-up tilts. It appears that descending projections from higher brain centers decrease the responsiveness to labyrinthine inputs of neurons in the pathways regulating SNA.

It has been postulated that regions of the cerebellum, including the posterior cerebellar vermis (the uvula, lobule IX) are components of the neural circuitry that adjusts the sensitivity of RVLM neurons to particular sensory inputs, including vestibular signals (see Figure 2). Purkinje cells in the posterior cerebellar vermis project to the caudal vestibular nucleus complex, which has monosynaptic and polysynaptic connections with the RVLM (Angaut and Brodal, 1967; Precht et al., 1976; Shojaku et al., 1987; Walberg and Dietrichs, 1988; Paton et al., 1991; Sugiyama et al., 2011; Holstein et al., 2011a), providing a pathway through which the uvula could modulate SNA. A disynaptic link also connects the uvula and NTS that may participate in adjusting the gain of baroreceptor responses (Paton et al., 1990, 1991). Electrical or chemical stimulation of the uvula produces changes in RVLM unit activity (Silva-Carvalho et al., 1991) and blood pressure (Nisimaru and Yamamoto, 1977; Bradley et al., 1987; Henry et al., 1989; Paton and Gilbey, 1992). In addition, lesions of the uvula produced a three-fold increase in the 10-Hz rhythm in SNA, but had little effect on the cardiac-related rhythm (Barman and Gebber, 2009). Thus, the uvula appears to play a specific role in controlling SNA, and does not simply modulate the excitability of brainstem neurons that regulate SNA. However, additional experiments will be needed to more precisely define that role.

## Moving forward: future applications of neurophysiological approaches to ascertain the neural mechanisms that control SNA

As discussed above, neurophysiological approaches have provided a number of important insights into the brainstem and spinal cord mechanisms that contribute to regulating SNA. The use of such approaches in decerebrate and anesthetized animal preparations revealed the areas of the brainstem that play key roles in generating rhythmic SNA and reflex-mediated changes in SNA. Experiments in anesthetized animals also showed that a variety of supratentorial brain regions, including areas of cerebral cortex, provide inputs to NTS and the RVLM (Verberne et al., 1997; Verberne and Owens, 1998), but little is known about the roles that these areas play in regulating SNA. While microneurography studies in humans have shown that stress alters MSNA (Anderson et al., 1991; Callister et al., 1992; Carter et al., 2005; Carter and Lawrence, 2007; Carter and Ray, 2009), and experiments in both humans and animals revealed that the dynamic properties of the baroreceptor reflex are altered by stress and particular behavioral repertoires such as the defense reaction (Del Bo et al., 1985; Grippo et al., 2008; Grippo and Johnson, 2009), little is known about how these conditions affect the processing of signals by the brainstem circuitry that controls SNA. One of the few studies that characterized the activity of RVLM neurons in conscious animals suggested that expression of cardiac-related activity by particular neurons could be labile, and dependent on the animal's cognitive state (Barman et al., 2011). However, considerable additional research is needed to appreciate the effects of stress and emotions on the firing rate and integration of signals by the brainstem neurons that modulate SNA. Similarly, our understanding is quite rudimentary of the neural mechanisms responsible for feedforward cardiovascular responses such as the defense reaction and central command.

Hence, despite considerable progress in understanding relatively simple brainstem pathways that generate rhythmic SNA and produce reflex-mediated changes in SNA, much is left to be learned. While some insights will be achieved through experiments in humans that combine microneurography with functional imaging (Critchley et al., 2011; Macefield et al., 2013), as with other fields of neuroscience recordings of neuronal activity in awake, behaving animal models will be needed to address many of the remaining scientific questions. Optimally, such experiments should also include the chronic recording of SNA, or at least some measure of cardiovascular responses to changes is SNA (e.g., blood pressure and heart rate). Most contemporary neurophysiological studies entail recordings in conscious animals or humans, as it is appreciated that the integration of information from the environment varies profoundly in accordance with behavioral state (Schroeder et al., 2010). It is now well-established that the processing of a variety of sensory signals (olfactory, gustatory, auditory, somatosensory, visual, vestibular) is dependent on whether the inputs are encountered during an ongoing behavior or are imposed when an individual is inactive (Schroeder et al., 2010). For example, responses of neurons in rodent somatosensory cortex to whisker movements differ when the whisker is manipulated in a stationary animal and during active exploration of the environment (Castro-Alamancos and Bezdudnaya, 2015). The responses of brainstem neurons to sensory inputs can also vary depending on whether those inputs are elicited by imposed or active movements. For instance, some vestibular nucleus neurons are activated when an unexpected change in head position occurs, but not when an individual voluntarily moves their head, despite the fact that the inputs from the inner ear to the vestibular nuclei are equivalent under the two situations (Cullen et al., 2011). It is yet to be determined whether responses of NTS and RVLM neurons to baroreceptor and other sensory inputs are similarly context-dependent.

Experiments that incorporate the recording of responses of brainstem neurons that regulate SNA in classical conditioning paradigms and delayed response tasks could also be very useful. Monitoring changes in RVLM neuronal activity during tasks in which animals are rewarded for delaying a motor response following a cue could provide insights into the mechanisms of central command. Unlike studies of sensory and motor physiology, experiments considering the control of SNA have rarely been conducted using non-human primates. However, future use of non-human primate models may be needed to permit the sophisticated behavioral paradigms required to decipher the context specificity in processing of signals by neural pathways that control SNA.

Finally, use of recently-invented experimental paradigms such as optogenetics will also be helpful to discover the physiological role of descending projections from supratentorial areas to NTS and RVLM, particularly if those techniques are coupled with the recording of SNA and/or firing rates of brainstem neurons. For example, a recent study by Wenker et al. (2017) used ArchaerhodopsinT3.0 loss-of-function optogenetics to clarify the role of RVLM C1 neurons in intact, unanesthetized rats. They showed that these neurons have a very low level of activity at rest but are activated by hypoxia and baroreceptor denervation; also the activity of these C1 neurons is important for the maintenance of blood pressure under conditions of anesthesia. Combining neurophysiological recordings with behavioral paradigms could also be very useful to address a number of key questions. For example, it would be useful to compare the responses of RVLM neurons to baroreceptor and noxious stimuli in normal animals with those who have experienced acute and chronic stress. Neurophysiological approaches in conscious animals are the best methods to discern how stress alters the processing of signals by brainstem neurons that control SNA, potentially providing insights into new treatment paradigms for psychiatric conditions.

## Summary and conclusions

Thousands of studies have entailed the monitoring of SNA in conscious or anesthetized humans, as well as conscious, anesthetized, or reduced (e.g., decerebrate) animal preparations. Use of each of these paradigms has strengths and weaknesses, and by mainly utilizing anesthetized or decerebrate preparations it was possible to determine the key areas of the brainstem that are responsible for generating rhythmic SNA, including cardiac-related activity that is related to the baroreceptor reflex. However, reliance on anesthetized or decerebrate preparations has been less successful in defining the role of multisynaptic connections that modulate SNA, including those arising in the cerebellum and supratentorial areas such as the hypothalamus, amygdala, and prefrontal and insular cortex. Future neurophysiological experiments in awake, behaving animal models will be required to delineate the neural mechanisms that contribute to adjusting SNA during stress and emotional states, and produce feedforward and anticipatory cardiovascular responses that occur during movement and specific behaviors.

## Author contributions

All authors listed have made a substantial, direct, and intellectual contribution to the work, and approved it for publication.

### Conflict of interest statement

The authors declare that the research was conducted in the absence of any commercial or financial relationships that could be construed as a potential conflict of interest.
